# Supplementary material for: Complexome profiling of the Chlamydomonas psb28 mutant reveals TEF5 as an early PSII assembly factor
Source: Plant Cell. 2025 Mar 17;37(6):koaf055. doi: 10.1093/plcell/koaf055 (PMC12164590; doi:10.1093/plcell/koaf055)
Supplement: koaf055_Supplementary_Data [file koaf055_supplementary_data.zip › psb28_interactive.zip!/plots/intensity/qualityPlots/peptides/_Cre10.g430000.t1.1.html]

### Cre10.g430000.t1.1

Trivial name:   
SeqCoverage [median]: 22.00  
Mass: 25.32 kDa  
  
 

| Sequence | MissedCleavage | Mass [Da] | wtIntensity | muIntensity | Score |
| --- | --- | --- | --- | --- | --- |
| AAFSAPAPAPR | 0 | 1054.56 | 53630.50 | 74625.69 | 186.91 |
| AAVTAAPAPAVEAEAPEAEPK | 0 | 1989.01 | 38302.63 | 59367.72 | 164.56 |
| IRAAVTAAPAPAVEAEAPEAEPK | 1 | 2258.19 | 877.03 | 5623.75 | 71.65 |
| NPSAGKVEVEEAAPK | 1 | 1524.78 | 48380.93 | 77436.53 | 165.34 |
| SILQER | 0 | 744.41 | 15764.64 | 40674.16 | 131.04 |
| SILQERNPSAGK | 1 | 1298.69 | 0.00 | 1091.99 | 43.54 |
| SILQERNPSAGKVEVEEAAPK | 2 | 2251.18 | 10579.96 | 26248.79 | 127.10 |
| SILQERNPSAGKVEVEEAAPKVSK | 3 | 2565.38 | 0.00 | 1117.86 | 56.77 |
| VEVEEAAPK | 0 | 970.50 | 9055.97 | 13283.05 | 135.83 |
|  |  |  | 176591.6 | 299469.5 |  |
